# Effect of Ultrasound and Osmotic Dehydration as Pretreatments on the Infrared Drying of Banana Slices

**DOI:** 10.17113/ftb.62.03.24.8409

**Published:** 2024-09

**Authors:** Fernanda Rezende Abrahão, Jefferson Luiz Gomes Corrêa, Arlley de Brito Magalhães Sousa, Paula Giarolla Silveira, Renata Nepomuceno da Cunha

**Affiliations:** 1 Department of Food Science, Federal University of Lavras, 37.200-900 Lavras, MG, Brazil; 2 Laboratory of Chemical Engineering, University Center of Patos de Minas, 38.702-054 Patos de Minas, MG, Brazil

**Keywords:** drying kinetics, mathematical modeling, food enrichment, isomaltulose, water loss

## Abstract

**Research background:**

There is a growing interest in foods with added nutritional value and extended shelf life. This study investigates the use of infrared technology in the drying of banana slices to improve their stability and quality by minimizing moisture content and water activity.

**Experimental approach:**

The drying experiments were carried out at a temperature of 70 °C, using the following pretreatments: ultrasound-assisted (UA) immersion in water for 20 and 30 min, osmotic dehydration (OD) and ultrasound-assisted osmotic dehydration (UAOD) for 20, 30 and 40 min. The osmotic process consisted of immersing the samples in the isomaltulose solution (40.0 g/100 g deionized water) for 60 min.

**Results and conclusions:**

All mathematical models used to describe the drying process showed a good fit with high R^2^ values (>0.98) and low value of the relative mean error E (%), the sum of squared error and the root mean squared error. The Fick’s diffusion coefficient (*D*_eff_) was higher for the samples previously treated with ultrasound for 20 and 30 min. The ultrasonic treatment resulted in shorter drying times with a reduction in average time of up to 29 %. OD was not efficient in reducing drying time, resulting in samples with lower drying rates. The samples treated with ultrasound showed less isotropic shrinkage and better color parameters. The osmotic process resulted in samples with greater rehydration capacity.

**Novelty and scientific contribution:**

The impregnation of a carbohydrate with low glycemic index in banana slices was achieved by the osmotic pretreatment, resulting in a new food product with attractive nutritional properties. This advancement represents not only a significant step in the development of functional foods, but also a major innovation in terms of processing technologies. The OD was combined with infrared drying, a method known for its superior drying rates, high heat transfer coefficient and energy efficiency. The synergy of these promising techniques not only shortens the processing times but also ensures more uniform dehydration of food products, resulting in end products that not only maintain but also optimize their nutritional value. These advances offer innovative solutions to improve food quality and also minimize environmental impact through low-energy technologies such as ultrasound and infrared treatment.

## INTRODUCTION

Drying is an important step in the food industry and a process for removing moisture from food characterized by the simultaneous transfer of heat and mass (*1*, *2*). Despite the advantage of extending the shelf life of food, drying can lead to some undesirable changes in the product, such as changes in the color, texture and degradation of nutrients (*2*, *3*). Therefore, the choice of drying method and the conditions are essential to obtain a good quality product (*2*-*4*). Convective drying is the most commonly used method. However, due to its low energy efficiency, it requires long drying time at high temperatures, which usually negatively affects the physicochemical properties of the final product. The use of new technologies, such as infrared radiation, can significantly increase drying rates and improve the quality of the product (*4*).

Infrared drying is a technology characterized by higher drying rates, a high coefficient of heat transfers and high energy efficiency. As a result, shorter process times and more uniform drying are obtained (*4*-*6*). Infrared radiation wavelength ranges between 0.78 and 1000 μm, and when drying food (3 to 6 μm), the radiation energy is converted into heat, which contributes to the dehydration of the product (*4*). In addition, the infrared energy is transferred from the heating source to the sample without heating the surrounding air, which heats the food quickly and uniformly (*5*). Infrared drying has already been studied in the literature for various food and agricultural products, such as turmeric root (*4*), banana slices (*7*) and ginger (*2*), among others. It is a technology that provides interesting results, but it is important to emphasize that its application is still limited to pilot and laboratory scale and requires greater energy and feasibility analyses for applicability on an industrial scale (*4*).

In addition, it is worth noting that higher infrared power can increase the drying rate, but higher temperatures can reduce the quality of the final product (*5*). In this way, the use of pretreatments before drying can effectively enhance the heat and mass transfer, reduce the damage to food and improve its quality (*5*). The main treatments commonly used in the literature include ultrasound (*8*-*11*) and osmotic dehydration (*12*, *13*).

Osmotic dehydration (OD) is a method that aims to partially remove moisture from the plant tissue by immersing the food in hypertonic solutions of salts and/or sugar (*13*, *14*). It is a technique that is very useful in the development of new products, as it increases the nutritional and functional value of the food without significantly altering its integrity (*8*). Before drying, OD can lead to higher drying rates and consequent reduction in process time. Reducing the exposure of food to high temperatures can lead to an improvement in its quality and acceptability, and also to a process with lower energy consumption (*14*, *15*). From a technological point of view, the OD can be an alternative for the incorporation of solutes with important nutritional properties, such as isomaltulose, a carbohydrate with a low glycemic index, prebiotic potential and recognized non-cariogenic properties (*16*).

The use of ultrasound aims to increase mass transfer rates, both in osmotic dehydration and in a subsequent drying process (*17*). In this technique, the food cut in pieces is immersed either in water or in a hypertonic aqueous solution, to which the ultrasonic energy is applied. The application of ultrasonic pretreatment increases the effective diffusivity of water in the food and results in a faster drying (*18*, *19*). The increase in the effective diffusivity of water can be attributed to the formation of microscopic channels by the ultrasonic waves (*17*). In addition, it is worth mentioning that ultrasonic treatment does not introduce soluble solids into the sample when only distilled water is used as a solvent (*20*).

The present study aims to evaluate the influence of ultrasound and osmotic dehydration on the infrared drying of bananas. The use of the pretreatments has the priority of reducing the processing and drying time, saving energy and improving the quality of the dried banana slices. To evaluate the drying kinetics, different mathematical models were applied to fit the data and evaluate the drying parameters. The characterization of the samples was based on the evaluation of shrinkage, water activity, color and the ability to rehydrate.

## MATERIALS AND METHODS

**Fig. S1:**
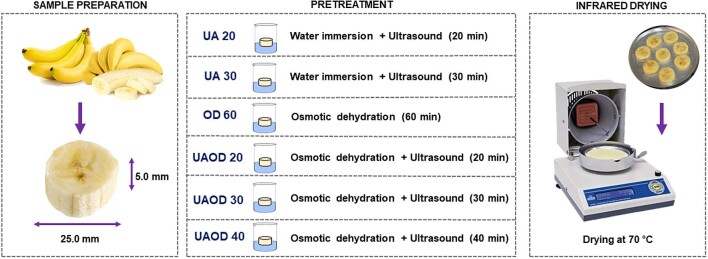
Schematic representation of sample preparation, pretreatments and infrared drying

In the present study, ultrasound alone and/or in combination with osmotic dehydration was used as a pretreatment in the infrared drying of banana slices (Fig. S1). The application of ultrasound to the banana slices immersed in water aimed to destabilize the cells in order to facilitate the subsequent drying process. The objective of including osmotic dehydration in the process was to increase the water loss of the samples and solutes with low glycemic index, in this case isomaltulose.

### Sample preparation

Bananas of the Prata variety (*Musa sapientum* L. cv. Prata) were purchased at the local market in the city of Patos de Minas, MG, Brazil. The fruits were selected based on their integrity and degree of maturation by coloring of the skin using the Von Loesecke scale (*21*). The bananas were selected in stages 3 and 4 (green/yellow). The fruits had a soluble solids content of (22.6±1.0) g/100 g and an initial moisture content of (2.7±0.2) kg/kg of sample on dry mass basis. The samples were disinfected with chlorinated water (200 mg/L) for 10 min, peeled manually and cut transversally into 0.5 cm thick slices with a diameter of 2.5 cm using a mold and a special stainless steel cutter.

### Pretreatments

Three different conditions were chosen for the processing of banana slices and the evaluation of their influence on infrared drying: immersion in deionized water in an ultrasonic bath, osmotic dehydration (OD) and ultrasound-assisted osmotic dehydration (UAOD). The experimental units consisted of eight banana slices placed in beakers containing deionized water or isomaltulose solution (40 g/100 g deionized water) with the ratio (*m*(liquid):*m*(sample))=4:1 (*8*, *22*). Commercial isomaltulose (Palatinose, Beneo, Mannheim, Germany) was used to prepare the osmotic solution.

In the ultrasonic pretreatment, the samples were exposed to ultrasonic energy in an ultrasonic bath (model 03502; Quimis, São Paulo, Brazil), with immersion times determined in preliminary studies. The experimental conditions applied were power of 70 W, frequency of 40 kHz and operating temperature of (25.0±3.0) °C. The volumetric power of the ultrasound was (7.9±0.6) W/L, determined by the calorimetric method (*19*). After the immersion time, the samples were removed from the beakers and the water on their surface was dried with absorbent paper.

For osmotic dehydration, the samples were immersed in concentrated isomaltulose solutions at room temperature (23.2 °C) without stirring. After 60 min, the samples were removed from the osmotic solution, placed on absorbent paper to remove the excess solution, weighed, and moisture content and soluble solids were analyzed. When used with ultrasound, the osmotic process (UAOD) was carried out with the device under the same conditions as the ultrasonic pretreatment. Prior to the osmotic process, the physicochemical properties of the isomaltulose solution were determined. The density was measured with a pycnometer at room temperature and the viscosity was determined using a rotary viscometer (model Q860A21; Quimis) with a concentric cylindrical sensor. The following values were obtained: pH=(7.55±0.11), *ρ*=1110 kg/m^3^ and *η*=(3.1±0.4) mPa·s.

The mass and moisture content of the samples were used to calculate the response variables of the experiment: water loss and solid gain, according to the following equations, respectively (*20*):


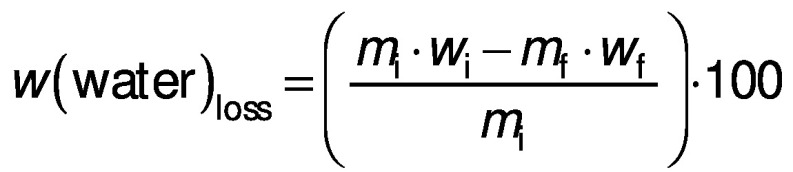
 /1/


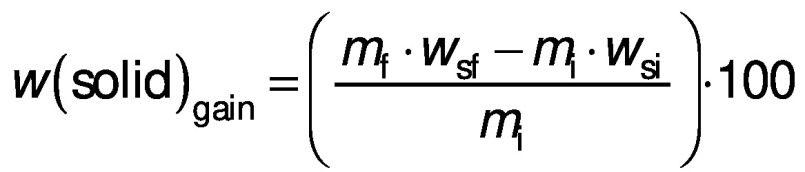
 /2/

where *m*_i_ and *m*_f_ are the initial and final mass of the sample (kg), *w*_i_ and *w*_f_ are the initial and final moisture content of the sample on a wet basis (kg/kg), and *w*_sf_ and *w*_si_ are the final and initial total solid content of the sample on a dry mass basis (kg/kg). All experiments were carried out in triplicate and the values given are based on the determined average values.

### Infrared drying

After the pretreatments, the banana slices were placed on aluminum trays (diameter 110 mm) and then dried. The control and pretreated samples were dried in a dryer with an infrared radiation source (model IV 2500; Gehaka, São Paulo, Brazil) at 70 °C for 360 min. The device contains a balance with a precision of 0.001 g and an automatic data collection system. The radiation source with an infrared power of 300 W was located at a fixed distance of approx. 15 mm from the samples.

The moisture mass fraction of the samples during the drying process was determined gravimetrically from the initial mass of the sample (before drying) and its mass at each time interval. The drying kinetics was analyzed by observing the drying curves for each condition. All drying experiments were performed with three replicates.

### Drying rate

The drying rate was determined from the moisture content of the samples (on a dry mass basis), according to the following equation (*23*):


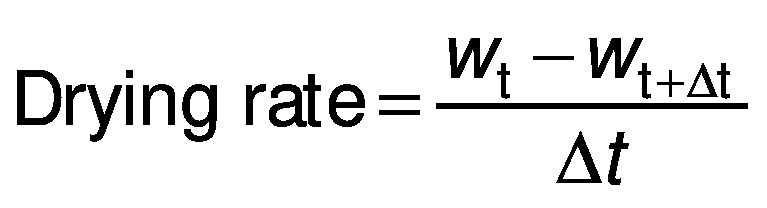
 /3/

where drying rate is in kg/(kg·min), *w*_t_ and *w*_t+Δt_ are the moisture content on dry mass basis (kg/kg) at drying time *t* (min) and *t+*Δ*t* respectively, and Δ*t* is the time interval (min).

### Mathematical modeling

The experimental data obtained during drying were fitted with three different equations for thin layer drying, *i.e.* logarithmic model (*24*) (Eq. 4), Page model (*25*) (Eq. 5) and Wang and Singh model (*26*) (Eq. 6):


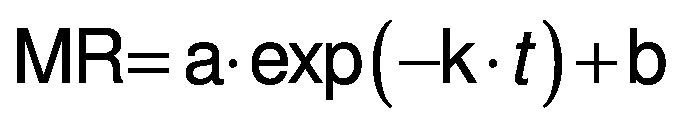
 /4/


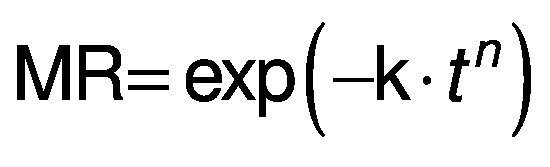
 /5/


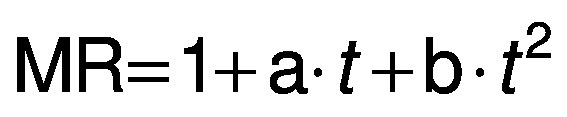
 /6/

where MR is the moisture ratio (dimensionless), *t* is the drying time (min), and a, b, n and k are empirical constants and coefficients of the drying equations.

The moisture ratio (MR) of the samples during the experiments was calculated using the following equation:


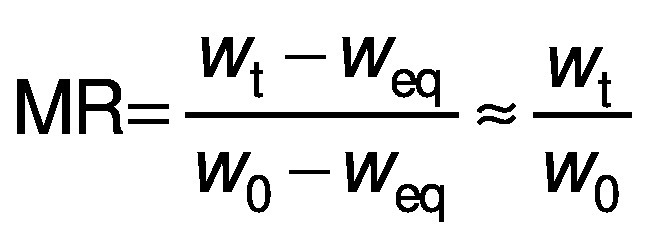
 /7/

where *w*_t_, *w*_0_ and *w*_eq_ are the moisture content during drying, the initial moisture content and the equilibrium moisture content on dry mass basis (kg/kg), respectively (*27*). Since the value of the equilibrium moisture content (*w*_eq_) is much lower than the value of the initial moisture content (*w*_0_) and the moisture content during drying (*w*_t_), it was assumed that this value was close to zero under the drying conditions studied.

### Effective moisture diffusivity

The experimental data were fitted to the unidirectional diffusion model to estimate the effective diffusivity of the water transferred during the drying process. Fick's law for unidirectional diffusion was applied (*27*, *28*):


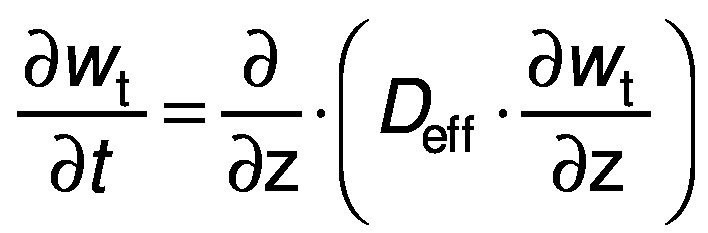
 /8/

where *w* is the moisture mass fraction on dry mass basis (kg/kg) at time *t*, *D*_eff_ is the effective diffusivity (m^2^/s), z is a general coordinate (m) and *t* is time (s). Considering the shape and dimensions of the banana slices, it could be assumed that diffusion did not occur in the angular and radial directions, so the characteristic length of the sample was *l*(plate)=2 m (half thickness). For such a geometry, the value of *D*_eff_ was determined according to the following equation:


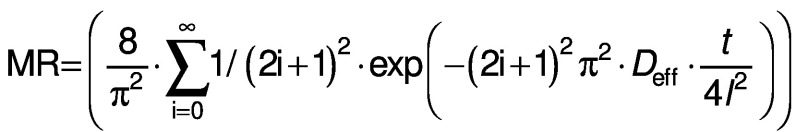
 /9/

where MR is the dimensionless water content. The initial condition is a uniform initial amount of water or solid, X(_z, 0_)=X_o_. The boundary conditions are the concentration symmetry 
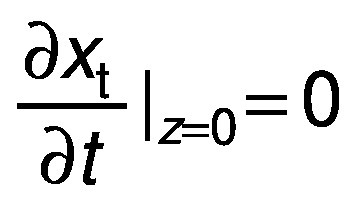
 and the equilibrium content on the surface X(_l, t_)=X_eq_ (*26*, *27*).

### Volumetric shrinkage

The volume (*V*) of the samples was calculated from the thickness (*δ*) and the average of the diameter measurement (*d*) at three positions for each slice. The diameter and thickness were measured with a calibrated digital caliper. Three samples were analyzed for each treatment during drying. The volumetric shrinkage was evaluated in the radial (SR) and the longitudinal (SL) directions, taking into account the beginning and the end of the drying process (*28*), and calculated using the following equations:


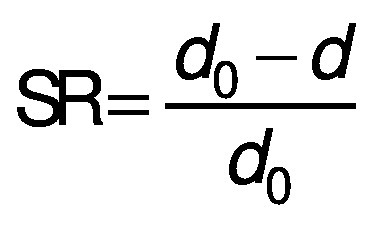
 /10/


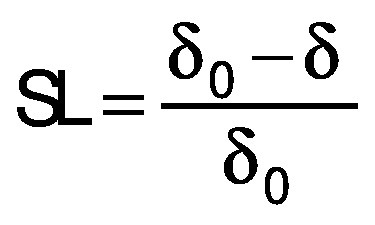
 /11/

where *d*_0_ is the diameter of the sample before drying, *d* is the diameter of the dried samples, *δ* is the thickness of the dried sample and *δ*_0_ is initial thickness of the sample. The shrinkage coefficient (SC) indicates how much the volume (*V*) of the sample decreased during the drying process. To determine the volume, the samples were considered as a disk. The diameter and thickness of the samples were determined using a calibrated digital caliper. This coefficient was determined according to the following equation (*28*):


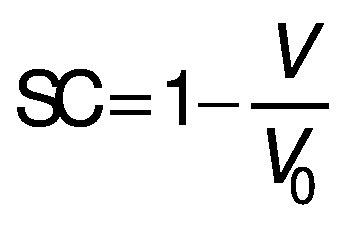
 /12/

The isotropic shrinkage (SI) was determined from the ratio between the radial shrinkage and the longitudinal shrinkage, as shown in the following equation:


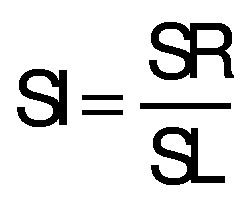
 /13

### Moisture content and water activity

The moisture content of the dried samples was determined gravimetrically in a vacuum oven at 70 °C until constant mass (*29*). The water activity (*a*_w_) was determined in a hygrometer (model Aqualab 3TE; Decagon Devices, Inc., Pullman, WA, USA). Analyses were carried out in triplicate.

### Colorimetric analysis

The color of the surface of the dried samples was analyzed using a digital colorimeter (model CR-400; Minolta, Osaka, Japan). Measurements were taken at different positions of each sample. The CIELAB coordinate system (L*, a* and b*) was measured with illuminant D65 (*27*). Chroma (C*) and hue (*h*°) were calculated according to the following equations:


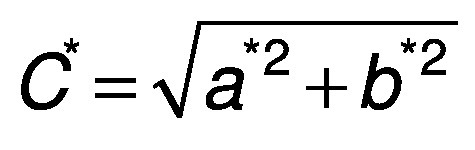
 /14/


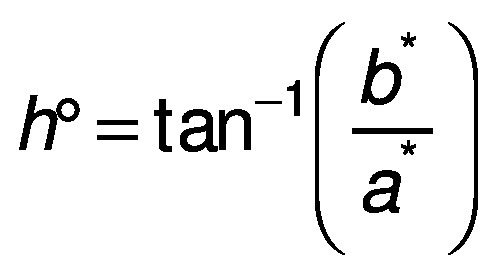
 /15/

### Rehydration capacity

The evaluation of the water absorption capacity of the dry samples was determined by the rehydration capacity (RC). The dried samples (approx. 1 g) were immersed in 150 mL of deionized water at (25±2) °C. After 135 min of immersion, the samples were removed from the water, placed on absorbent paper to remove the excess water and weighed. The change in the mass of the samples was used to evaluate the rehydration process according to the following equation (*27*):


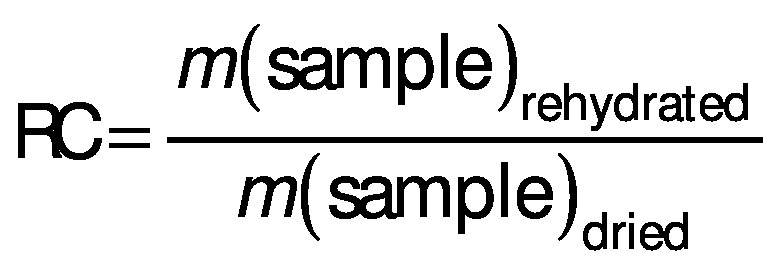
 /16/

All tests were performed in quadruplicate and mean values were used for calculations.

### Statistical analysis

The parameters and constants of the empirical equations were estimated by correlating the mathematical models with the experimental data using a quasi-Newton method for nonlinear regression at a 5 % significance level with Statistica 7.0® software (*30*). The evaluation of the mathematical models was based on the highest coefficient of determination (R^2^) and the lowest value of the relative mean error (E in %) (Eq. 17), where *m*_i_ is the experimental value, *m*_p_ is the predicted value and *N* is the sample size. Other statistical parameters such as the sum of squared error (SSE) (Eq. 18) and the root mean square error (RMSE) (Eq. 19) were also used to evaluate and compare the fit.


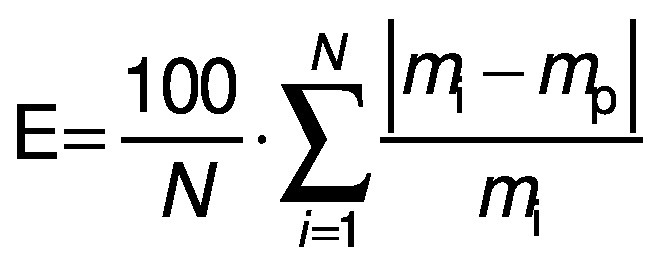
 /17/


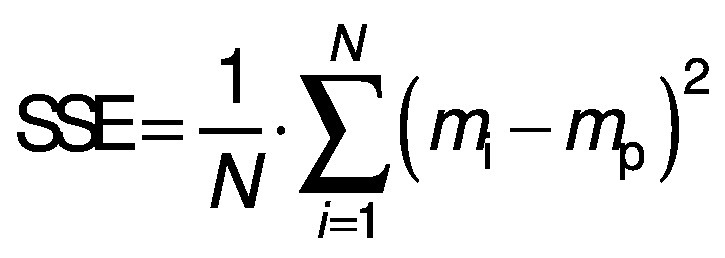
 /18/


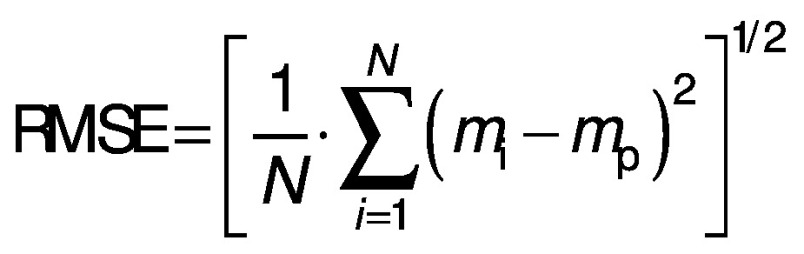
 /19/

ANOVA was used to evaluate the results of the qualitative analyses, followed by the Tukey’s test, both with a significance level of 5 %. The analyses were performed with the software Sisvar v. 5.8 (*31*).

## RESULTS AND DISCUSSION

### Effect of the pretreatments on the banana slices

The effect of the pretreatments on the water loss, solid gain and final moisture (X) of the samples is shown in Table 1. During ultrasonic pretreatment, the samples lost solids and gained water (negative sign). This result is due to the fact that deionized water is hypotonic compared to the food, creating concentration gradients between the two media that favor the transfer of solids from the fruit into the water and the transfer of water from the medium into the fruit. As a result of this behavior, the final moisture content of the samples increased by 47.01 and 52.08 % when ultrasound was applied for 20 and 30 min, respectively. Similar results were observed in other studies (*8*, *20*, *22*, *32*-*34*).

**Table 1:** Water loss, solid gain and moisture content of samples after the application of pretreatments

**Treatment**	***w*(water)_loss_/%**	***w*(solid)_gain_/%**	***w*(moisture)/(kg/kg)**
**UA 20**	-14.5±1.5	-2.3±0.4	4.0±0.1
**UA 30**	-17.5±1.3	-3.0±0.3	4.1±0.2
**OD 60**	5.2±0.9	4.1±0.9	2.4±0.1
**UAOD 20**	1.1±0.3	1.5±0.1	2.4±0.1
**UAOD 30**	2.4±0.6	3.4±0.2	2.4±0.3
**UAOD 40**	2.9±0.4	3.7±0.2	2.4±0.1

Results are presented as mean value±standard deviation, *N*=3. UA 20 and 30=treatment with ultrasound for 20 and 30 min, respectively, OD 60=osmotic dehydration for 60 min, UAOD 20, 30 and 40=ultrasound-assisted osmotic dehydration for 20, 30 and 40 min

The use of ultrasonic energy promoted the removal of native solutes from the fruit (Table 1). This behavior is important because it shows that ultrasonic technology efficiently removes sugars and solids from fruit and can be used in the production of foods with reduced calorie content (*22*, *32*). During exposure to ultrasound, the samples are subjected to an alternating cycle of compression and expansion, which can lead to the formation of microchannels that cause the loss of water and solutes from the fruit slices to the liquid medium (*20*, *32*). In addition, it can be observed that both water loss and solid gain values increased with the increase in the ultrasound duration in the pretreatments. For the UAOD in particular, the increase in these values indicates that the use of the technology improves the mass transfer processes. A similar result was observed in a study with cranberries, in which the increase in water loss and solid gain was attributed to cavitation, which increases cell permeability and accelerates molecular diffusion (*34*).

The osmotic process with isomaltulose led to a solid gain in the fruit and a loss of water from the fruit to the medium. The loss of water from the banana slices was already expected, as the application of hypertonic solutions leads to a high concentration gradient, which increases the mass transfer processes. It was also observed that the percentage of water loss during osmotic dehydration for 60 min was higher than that of solid gain. This behavior is due to the selectivity of the cell membrane, which allows the transport of smaller molecules, such as water, but restricts the transport of larger molecules, such as isomaltulose (*22*).

During the osmotic process, several variables influence the solid gain and water loss of the samples, such as the process time and the concentration of the osmotic solution. Traditionally, the optimal process conditions are those that result in the highest water loss and the lowest solid gain. However, from a nutritional and sensory point of view, a lower inclusion of solids in the osmotic dehydration is interesting (*22*). Furthermore, as the osmotic process does not require high temperatures or specialized equipment, it could be a good alternative for the impregnation of fruit and vegetables with low-caloric solutes, such as isomaltulose.

### Evaluation of the drying kinetics

**Fig. 1:**
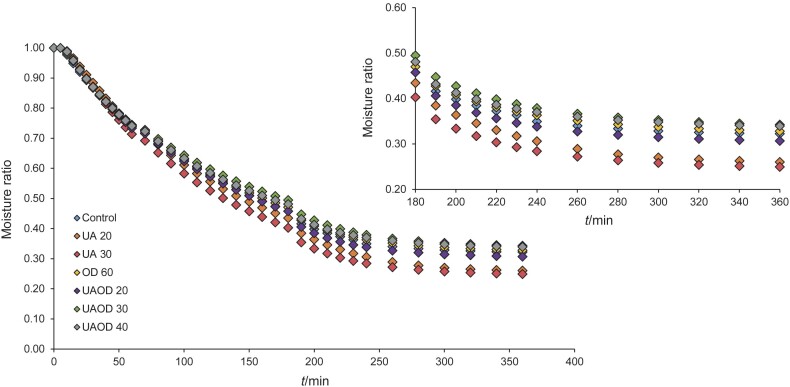
Variation of the moisture ratio with the drying time of banana slices subjected to different pretreatments. UA 20 and 30=treatment with ultrasound for 20 and 30 min, respectively, OD 60=osmotic dehydration for 60 min, UAOD 20, 30 and 40=ultrasound-assisted osmotic dehydration for 20, 30 and 40 min

**Fig. 2:**
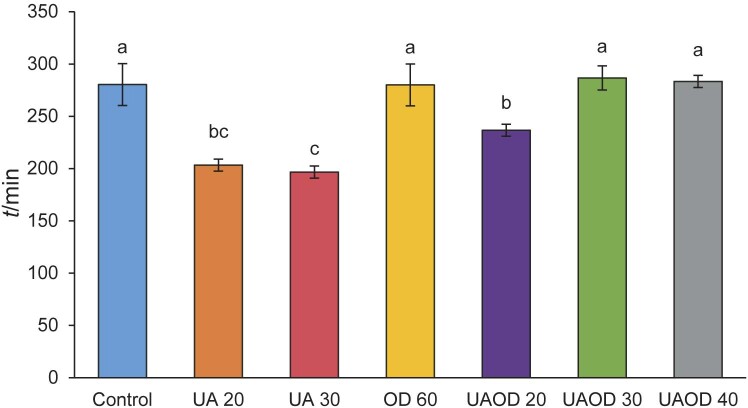
Drying time to reach 25 % moisture (wet basis) of bananas with and without the use of pretreatments. Mean values followed by different letters differ significantly (p≤0.05) according to Tukey's test. UA 20 and 30=treatment with ultrasound for 20 and 30 min, respectively, OD 60=osmotic dehydration for 60 min, UAOD 20, 30 and 40=ultrasound-assisted osmotic dehydration for 20, 30 and 40 min

To evaluate the influence of the pretreatments on the kinetics of infrared drying of banana slices, the dimensionless moisture ratio (MR) and its change during drying is shown in Fig. 1. The average drying times required by the samples to reach a 25 % moisture on wet mass basis are shown in Fig. 2.

Fig. 1 shows that the moisture ratio of the samples decreased with the drying time and exhibits an exponential decay. In the initial stage, the process is mainly controlled by the diffusion mechanism, with the moisture ratio dropping rapidly until the equilibrium moisture is gradually reached (*35*). In general, immersion in water using ultrasound as a pretreatment accelerated the drying process of the samples compared to the control and samples treated with osmotic dehydration. Compared to the control samples, the drying time of the ultrasound-treated banana slices was reduced by 27.48 and 29.8 % when they were treated with ultrasound for 20 and 30 min, respectively. In the case of infrared drying of pear slices, ultrasonic pretreatment resulted in shorter drying times and thus in a dry product with less quality loss (*3*). Similar results were observed in the convective drying of carrots (*33*), papaya (*32*, *36*), ginger (*15*), bitter melon (*11*) and bananas (*20*). When immersed in water with an ultrasonic bath, the cellular structure is disintegrated, either by the ultrasonic energy or by the transport of water into the cellular structure. This disintegration of the cellular microstructure facilitates the diffusion of water during the subsequent drying process (*15*, *33*), thereby reducing energy costs and improving the final quality of the dried product.

The use of osmotic dehydration as a pretreatment in the infrared drying of banana slices did not result in shorter average drying times compared to the control samples. The application of the osmotic process at times longer than 20 min, regardless of the use of ultrasound, resulted in longer average drying times and higher MR (Fig. 1). This result could be due to the formation of a layer of isomaltulose on the surface of the fruit after the osmotic process, which may have impeded the water flow, reduced the diffusivity and consequently increased the drying time (*9*). A similar result was observed in studies of osmotic dehydration of carrots (*9*), strawberries (*37*) and papaya (*32*).

### Drying rate of the infrared process

**Fig. 3:**
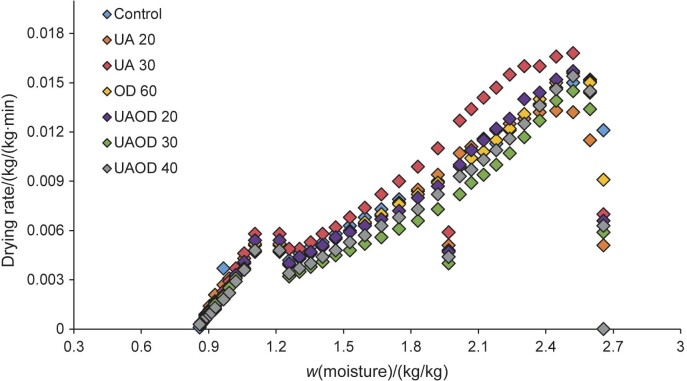
Drying rate of banana slices as a function of *their* moisture content. UA 20 and 30=treatment with ultrasound for 20 and 30 min, respectively, OD 60=osmotic dehydration for 60 min, UAOD 20, 30 and 40=ultrasound-assisted osmotic dehydration for 20, 30 and 40 min

The drying rate of the samples subjected to the different pretreatments is shown in Fig. 3. In the initial phase of drying, the banana slices had a high moisture content, resulting in high drying rate values. When the moisture content of the samples is significantly reduced over time, the drying rate decreases as it is difficult to remove the water (*1*, *35*). Furthermore, drying times with lower drying rates indicate that the surface of the product is no longer saturated with water and thus the drying rate is controlled by the diffusion of moisture from the interior of the food to the surface (*4*). An important point to emphasize is that as the infrared energy penetrates directly into the inner layer of the food, it is absorbed more by the molecules so that it is converted into thermal energy and consequently increases the drying rate. This is one of the main advantages of this technology, as it avoids energy loss and preserves the quality of the product (*35*, *36*).

Of all the pretreatments applied, the drying rate of the samples treated with ultrasound for 30 min showed the highest values throughout the process. Lower drying rates were observed in the samples subjected to osmotic treatment, especially those treated for 30 and 40 min (UAOD 30 and UAOD 40, respectively, Fig. 3). Macedo *et al*. (*1*) reported that the inclusion of solutes in osmotic processes increases the interaction of water with food, in which it affects the flow and diffusion of water. Shi *et al*. (*6*) mentioned that the monosaccharides present in banana can promote the formation of hydrogen bonds between the free water molecules and the hydroxyl groups. To break these bonds, a longer process time and more energy are required, which results in lower drying rates.

### Mathematical modeling of the drying kinetics

For the mathematical modeling, the experimental moisture contents (dry basis) throughout the drying process were converted into MR values ​​and adjusted for time. Three empirical models of thin layer drying were compared. The adjustment parameters, constants and the comparison criteria of the applied mathematical models are shown in Table 2. All mathematical models related to the experimental data showed a good adjustment and had high R^2^ values and lower values of SSE, E (%) and RMSE, indicating higher reliability of the model. The three mathematical models obtained R^2^ values between 0.993 and 0.999, low SSE values (<0.002), E (%) below 6.475 and low RMSE values (<0.051). For green (*19*) and ripe bananas (*38*, *39*), the mathematical modeling proved to be similar, highlighting the application of Wang and Singh’s empirical model. Models like logarithmic and Wang and Singh’s have a greater number of coefficients and determination parameters that provide a better description of the drying curve of banana slices.

**Table 2:** Parameters, coefficients of determination (R^2^), relative mean error (E), sum of squared error (SSE) and root mean square error (RMSE) of the mathematical models fitted to the moisture ratio data at different pretreatments

**Model**	**Parameter**	**R^2^**	**E/%**	**SSE**	**RMSE**
**Control**					
**Page**	k=0.008; n=0.868	0.994	0.618	6·10^-4^	0.025
**Wang and Singh**	a=−0.004; b=7·10^-6^	0.999	0.043	1.6·10^-4^	0.011
**Logarithmic**	a=0.788; b=0.007; k=0.234	0.998	0.092	1.8·10^-4^	0.013
**U**A** 20**					
**Page**	k=0.005; n=0.972	0.996	0.920	6·10^-4^	0.024
**Wang and Singh**	a=−0.005; b=7·10^-6^	0.998	6.475	0.002	0.051
**Logarithmic**	a=0.893; b=0.007; k=0.144	0.998	0.102	2.2·10^-4^	0.015
**U**A** 30**					
**Page**	k=0.007; n=0.945	0.995	1.114	7·10^-4^	0.027
**Wang and Singh**	a=−0.005; b=8·10^-6^	0.998	0.153	2.1·10^-4^	0.014
**Logarithmic**	a=0.881; b=0.007; k=0.157	0.998	0.126	2.4·10^-4^	0.015
**OD 60**					
**Page**	k=0.008; n=0.865	0.994	0.621	6·10^-4^	0.025
**Wang and Singh**	a=−0.004; b=7·10^-6^	0.999	0.030	1.5·10^-4^	0.012
**Logarithmic**	a=0.779; b=0.007; k=0.244	0.998	0.078	1.7·10^-4^	0.013
**UAOD 20**					
**Page**	k=0.008; n=0.892	0.994	0.670	7·10^-4^	0.026
**Wang and Singh**	a=−0.004; b=7·10^-6^	0.998	0.003	2.1·10^-4^	0.014
**Logarithmic**	a=0.813; b=0.007; k=0.215	0.998	0.130	2.5·10^-4^	0.016
**UAOD 30**					
**Page**	k=0.009; n=0.849	0.994	0.522	6·10^-4^	0.024
**Wang and Singh**	a=−0.004; b=7·10^-6^	0.998	0.117	2.3·10^-4^	0.015
**Logarithmic**	a=0.760; b=0.007; k=0.259	0.998	0.086	2·10^-4^	0.014
**UAOD 40**					
**Page**	k=0.009; n=0.849	0.993	0.595	7·10^-4^	0.026
**Wang and Singh**	a=−0.004; b=7·10^-6^	0.998	0.060	2.1·10^-4^	0.014
**Logarithmic**	a=0.762; b=0.007; k=0.262	0.998	0.091	2·10^-4^	0.013

a, b and n are the parameters and k is the constant of the mathematical models. UA 20 and 30=treatment with ultrasound for 20 and 30 min, respectively, OD 60=osmotic dehydration for 60 min, UAOD 20, 30 and 40=ultrasound-assisted osmotic de hydration for 20, 30 and 40 min

### Effective moisture diffusivity of the drying process

The effective diffusivity (*D*_eff_) values obtained for the banana slices ranged from 1.10·10^-11^ to 1.63·10^-11^ and were comparable to the values reported in the literature for dehydrated bananas (*8*, *19*, *28*, *37*-*39*). The banana slices immersed in water and treated with ultrasonic energy (for 20 and 30 min) had a higher *D*_eff_ value ((1.47±0.06)·10^-11^ and (1.63±0.09)·10^-11^, respectively) than the control ((1.26±0.08)·10^-11^) and other treatments, possibly due to the destabilization of the cells caused by cavitation. The application of ultrasound to unripe bananas resulted in a 4.8 % increase in effective moisture diffusivity (*19*). In papaya, the increase in diffusivity by the use of ultrasound was associated with the formation of microchannels that facilitate the movement of water molecules to the surface of the fruit (*32*). When combined with ethanol as a pretreatment, ultrasound promoted higher diffusivity values in bananas, with an increasing trend observed with increasing treatment time (*40*). In persimmons, ultrasound-assisted osmotic dehydration led to a significant reduction in drying time and thus to an increase in effective diffusivity values of up to 21 % (*41*).

The samples pretreated by osmotic dehydration, with and without the use of ultrasound, had lower diffusivity values (from 1.10·10^-11^ to 1.23·10^-11^). This can be explained by the physical and chemical changes in the banana slices during the incorporation of isomaltulose, which led to different drying rates and consequently to lower diffusivity value. The osmotic process promotes saturation of the surface of the samples with solute molecules, creating an additional resistance for the heat and mass transfer processes (*13*, *14*, *32*). Macedo *et al*. (*1*) also observed that samples to which isomaltulose was added tended to require a longer drying time. The authors correlated this with the incorporation of solutes during the osmotic processes, which increases the amount and strength of water bonds with the food and hinders moisture loss.

### Effect of the drying conditions in the volumetric shrinkage

Shrinkage is an important parameter of dried foods and a physical change related to the drying process and the conditions applied (*37*). The loss of water in combination with the high temperature causes stress to the cell tissue, changes its microstructure and promotes its shrinkage (*5*, *27*). The longitudinal shrinkage of the samples ranged between 0.22±0.09 and 0.50±0.05. The radial shrinkage ranged from 0.16±0.05 to 0.21±0.04, with no significant differences between the treatments. It can be observed that the longitudinal shrinkage was greater than the radial shrinkage. A similar behavior was observed in a study on convective drying of green and ripe bananas (*28*). According to the authors, this behavior is expected since the mass transfer occurs longitudinally due to the position of the heat source.

The shrinkage coefficient ranged from 0.5±0.1 to 0.70±0.01, with no significant differences between the treatments. The samples treated with osmotic dehydration, with and without the ultrasonic assistance, showed higher values of shrinkage isotropicity (between 0.58±0.09 and 0.8±0.2). This behavior can be attributed to the removed moisture and cellular stress due to the osmotic process and subsequent drying. As a result of the reduction in total drying time, the samples treated with ultrasound for 30 min showed a greater cell wall integrity and less shrinkage, with lower isotropic shrinkage values (0.4±0.1) than the samples treated with other methods. The application of ultrasonic energy makes the cellular microstructure more porous, allowing better diffusion of water and faster removal of moisture towards the surface. It is also important to note that the rapid evaporation of water triggered by drying can create a large flow of steam on the surface, which helps to prevent the collapse of the cell tissue and the shrinkage (*42*).

During drying, the removal of moisture promotes the reduction of the volume of the banana slices, as water is one of the main components of their composition. The shrinkage of the fruit increases with the volume of the removed water, as the higher the moisture content removed, the more contraction stresses are created in the structure (*18*). According to Corrêa *et al*. (*28*), the shrinkage of banana slices during convective drying is linearly related to the moisture ratio. Furthermore, it is important to emphasize that shrinkage can be influenced by several factors, such as the degree of fruit maturation, the variety and the drying conditions, which makes comparison with other studies in the literature difficult.

### Evaluation of the moisture content and the water activity

The final moisture mass fraction of the samples on dry mass basis ranged from 0.13 to 0.24 kg/kg, with no statistical difference (p>0.05) between treatments (Table 3). However, it should be noted that the banana slices that were subjected to osmotic treatment (osmotic dehydration and UAOD) had a lower final moisture content, which is important from a technological and conservation point of view.

**Table 3:** Final moisture mass fraction (on dry mass basis), water activity (*a*_w_) and color parameters of dried banana slices

**Code**	*w*_f_(moisture)/(kg/kg)	*a*_w_	Color parameter								
a*		b*		C*		L*		*h*°
**Control**	(0.22±0.02)^a^	(0.548±0.005)^a^		(6.4±1.2)^ab^		(20.7±2.4)^ab^		(21.7±2.2)^ab^		(52.6±4.8)^a^	(72.8±4.2)^a^
**U**A** 20**	(0.24±0.04)^a^	(0.545±0.009)^a^		(6.2±1.1)^ab^		(23.9±5.0)^ab^		(26.8±9.8)ª		(52.7±4.0)^a^	(75.1±3.4)^a^
**U**A** 30**	(0.23±0.07)^a^	(0.558±0.006)^a^		(7.1±0.7)ª		(24.8±3.1)ª		(25.8±2.8)^ab^		(53.9±3.2)^a^	(73.8±3.0)^a^
**OD 60**	(0.21±0.02)^a^	(0.561±0.005)^a^		(6.3±1.1)^ab^		(23.7±4.6)^ab^		(24.6 ±4.4)^ab^		(55.4±4.0)^a^	(74.7±3.7)^a^
**UAOD 20**	(0.17±0.05)^a^	(0.6±0.02)^a^		(6.4±2.1)^ab^		(20.3±2.0)^ab^		(21.2±1.7)^ab^		(56.6±7.0)^a^	(74.06±5.8)^a^
**UAOD 30**	(0.13±0.06)^a^	(0.54±0.01)^a^		(5.0±1.7)^b^		(18.9±2.6)^b^		(19.6±2.8)^b^		(58.3±8.0)^a^	(75.5±4.0)^a^
**UAOD 40**	(0.19±0.07)^a^	(0.54±0.01)^a^		(5.2±1.6)^ab^		(20.7±3.4)^ab^		(21.9±3.5)^ab^		(57.3±7.2)^a^	(75.7±6.3)^a^

Results are mean value±standard deviation, *N*=3. The same letter in the column indicates no significant difference between treatments according to Tukey's test (p<0.05). UA 20 and 30=treatment with ul­trasound for 20 and 30 min, respectively, OD 60=osmotic dehydra­tion for 60 min, UAOD 20, 30 and 40=ultrasound-assisted osmotic dehydration for 20, 30 and 40 min

Although there was no statistically significant difference (p>0.05), all pretreatments resulted in dry samples with *a*_w_ values below 0.6 (Table 3). This data is important as foods with high *a*_w_ are more susceptible to microbial growth and the resulting degradation (*37*). It is generally assumed that the highest food stability is achieved at reduced *a*_w_ values corresponding to the water content in the monolayer (*43*). In addition, in some treatments, the addition of isomaltulose altered the structure and cellular composition of the banana slices, promoting new interactions between the water molecules and the sample, which affected the water fugacity and consequently the *a*_w_ value (*1*). It is important to highlight that the osmotic dehydration can in some cases lead to products with higher *a*_w_, which could be related to the crystallization of solutes, especially sugars, after drying (*43*). This has been observed in studies with kiwiberry (*43*), strawberry (*12*) and papaya (*44*).

### Appearance of the dried bananas

The color of a food is an important parameter that influences its acceptability and also provides information about some aspects of its quality and properties (*1*, *15*, *27*). The values of the parameters L* (brightness/luminosity), a* (red/green), b* (yellow/blue), C* (chroma) and *h*° (hue) of the banana slices dried with infrared radiation under different conditions are shown in Table 3. The results show a significant difference (p<0.05) in the color parameters, suggesting that the color of the samples was altered by the applied pretreatments. The treatment with ultrasound for 30 min led to an increase in a*, b* and C* parameters, while the UAOD treatment for 30 min led to a decrease in the same parameters. The increase in a* value, which indicates a more reddish color, can be attributed to the darkening that occurs mainly during cell destabilization by ultrasound. Non-enzymatic processes, such as the Maillard reaction, occur during the drying process and can also lead to the formation of reddish and dark pigments (*11*, *39*). High b* values (+b*) indicate saturation of the yellow color, while low values ​​indicate leaching and degradation of pigments, which is due to the osmotic treatment of some samples (*14*, *44*). In addition, the incorporation of isomaltulose leads to the formation of a solid layer on the surface, which limits the contact between the samples and oxygen and thus reduces oxidation reactions and possible browning (decreased parameter b*) (*45*).

There were no statistically significant differences (p>0.05) in the values of the parameters L* and *h*°. The values of the L* parameter, which ranged from 52.6 to 58.3, could indicate darker samples, in which dark pigments, such as melanoidins, formed during the drying process (*1*, *39*). In general, shorter drying times and lower temperatures lead to higher L* values and the formation of fewer dark compounds. The osmotic dehydration can minimize thermal damage to the food coloring by preventing enzymatic browning. On the other hand, the use of highly concentrated solutions can cause changes in the cell wall, resulting in an undesirable appearance of the final product (*14*, *34*). Furthermore, some strategies can be used to minimize color variations. Immersing the banana slices in a citric acid solution prior to the osmotic process reduced excessive color changes and minimized the darkening of the final product (*46*). The same result was observed with banana slices dehydrated with infrared radiation (*7*).

### Rehydration capacity of bananas slices dried with infrared radiation

**Fig. 4:**
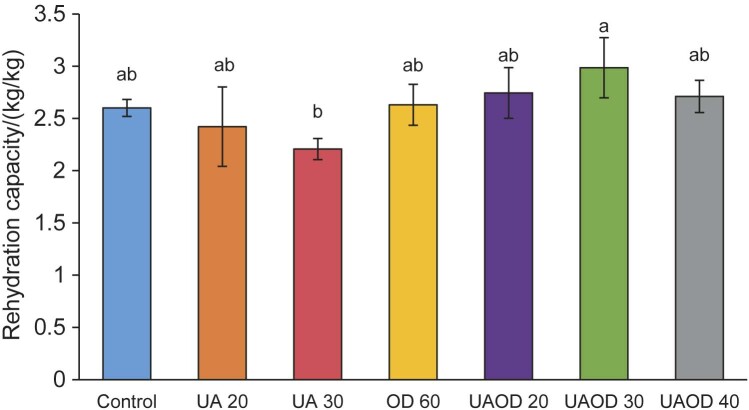
Rehydration capacity of banana slices dried with infrared radiation. Mean values followed by different letters differ significantly (p≤0.05) according to Tukey's test. US 20 and 30=treatment with ultrasound for 20 and 30 min, respectively, OD 60=osmotic dehydration for 60 min, UAOD 20, 30 and 40=ultrasound-assisted osmotic dehydration for 20, 30 and 40 min

Rehydration capacity is an important parameter in the evaluation of the quality of dehydrated products and is directly related to the drying conditions used and their effect on the structure and composition of the product (*10*, *17*). During rehydration, the food immersed in water undergoes several simultaneous changes, particularly in its moisture content, porosity and volume. The influence of the application of different pretreatments on the rehydration capacity of banana slices dried with infrared radiation is shown in Fig. 4. The rehydration capacity values ​​of the samples ranged from (2.2±0.1) to (2.9±0.38) kg/kg for all drying conditions analyzed. The application of ultrasonic energy facilitated water removal but also reduced the resistance to moisture absorption during the rehydration process. Lower rehydration capacity values ​​were observed for the samples treated with ultrasound for 30 min ((2.2±0.1) kg/kg) and higher rehydration capacity values ​​were observed for samples subjected to the osmotic process.

It is well known that higher infrared powers and higher temperatures lead to final products with a more porous structure, which directly affects their water absorption capacity (*47*). In addition, it is important to emphasize that the rehydration capacity and the amount of absorbed moisture depend on the degree of cellular and structural degradation caused by the application of pretreatments, such as ultrasonic energy (*10*, *17*, *42*). Although the ultrasound treatment causes rapid absorption of water by the samples, retention of the absorbed water can be impaired by structural damage and the formation of microchannels (*10*). Structural changes can lead to loss of water uptake by the cell cavities, which is proportional to the amount of water absorbed. High temperatures, both during drying and rehydration, reduce water absorption due to structural breakdown (*48*, *49*). Efficiently dried fruits and vegetables have a shorter drying time, less shrinkage and consequently better rehydration (*50*, *51*). Samples with minimal shrinkage after the drying process result in well-defined intercellular voids, which promotes an expressive rehydration rate (*52*).

The rehydration of foods previously dehydrated by osmotic dehydration is complex and depends on a number of factors. In the initial stage of rehydration, the solids are dissolved in the surface layer, which hinders the adsorption of water inside the sample. During the process, the cellular structure of the food determines the degree of rehydration (*48*, *53*). In addition, it is worth noting that osmotic dehydration affects the rehydration properties of the dried sample due to permeability and cellular disintegration as a result of osmotic stress, reducing the water absorption and retention capacity of the samples (*18*, *54*). In osmotic processes, the effect of the addition of sugar on the water absorption has already been observed in the literature (*12*, *41*, *42*, *52*, *53*, *55*). In their study, Cháfer *et al*. (*53*) reported the loss of solids and reduced water absorption by the samples treated with osmotic dehydration. This fact suggests that most of the solids absorbed during the osmotic step were lost during the rehydration process and their possible interactions with the fruit matrix did not contribute to the retention of these components. The authors conclude that osmotic dehydration is not recommended from a technological point of view when dried samples need to be rehydrated.

## CONCLUSIONS

The use of infrared radiation proved to be important to obtain dehydrated banana slices with reduced moisture content and water activity values (*a*_w_<0.6). In combination with osmotic processing, this enabled the production of a novel product, whose nutritional profile was enriched by the addition of isomaltulose, a low-glycemic carbohydrate. The new food can be an alternative for consumers with specific and restrictive diets. Although the technique promoted the impregnation of solutes in the fruit, it could not effectively reduce time and parameters of drying kinetics such as the effective diffusivity coefficient.

Osmotic dehydration is an important method for the preservation and processing of fruit and vegetables. It plays an essential role in preserving their chemical, sensory and functional properties. In addition, the process can also be associated with ultrasound and barrier technology through the preparation of solutions containing solutes with antimicrobial and functional properties.

The inclusion of ultrasonic energy as a pretreatment to the infrared drying reduced drying times by up to 29 %, improved diffusivity coefficients and minimized isotropicity, radial and longitudinal shrinkage. Additionally, this approach resulted in slices with vibrant colors and improved physical appearance.

Savings in drying time are important as they save energy and processing time and improve the properties of the final product. Shorter drying times mean less time exposed to high temperatures. The knowledge of these aspects is important to obtain a higher quality product and to optimize the method. In addition, further studies should be carried out to improve infrared drying using ultrasonic energy. The aim should be to adjust the power and energy required for drying, while maintaining the highest possible quality of the food. As the integration of food preservation techniques is refined to achieve the goals of reduced processing time and improved efficiency, the potential for industrial applications should be considered.
